# Medical College of Louisiana

**Published:** 1844-02

**Authors:** 


### MEDICAL COLLEGE OF LOUISIANA.

In a notice of this institution, contained in one of the Travelling Editorials of the Senior Editor, under date of March last, it is said: "The lectures are delivered in a rented house, but the Professors have begun the creation of a fund for the erection of an appropriate edifice." Since this was written, a commodious Medical Hall has been erected, and is now occupied by the Faculty. It gives us the greater pleasure to notice this fact, as it is the result solely of individual enterprise. Neither the State nor the city authorities lent their aid. Several physicians, however, unconnected with the institution, and some of the private citizens, showed a praiseworthy liberality, by valuable donations to the library and museum. We trust the expectations of all its friends may be realized, and that the Faculty may reap their reward both in emoluments and honors. C.

